# Transvenous lead extraction: Efficacy and safety of the procedure in female patients

**DOI:** 10.1016/j.hroo.2023.09.002

**Published:** 2023-09-12

**Authors:** Luca Segreti, Maria Grazia Bongiorni, Valentina Barletta, Matteo Parollo, Andrea Di Cori, Federico Fiorentini, Mario Giannotti Santoro, Raffaele De Lucia, Stefano Viani, Gino Grifoni, Luca Paperini, Ezio Sodati, Lorenzo Mazzocchetti, Antonio Maria Canu, Giulio Zucchelli

**Affiliations:** ∗ Department of Translational Research on New Technologies in Medicine and Surgery, University of Pisa, Pisa, Italy; † Second Division of Cardiovascular Diseases, Cardiac and Thoracic Department, New Santa Chiara Hospital, University of Pisa, Pisa, Italy

**Keywords:** Transvenous lead extraction, Sex differences, Female sex, Safety outcomes, Complication

## Abstract

**Background:**

Existing data on the impact of sex differences on transvenous lead extraction (TLE) outcomes in cardiac device patients are limited.

**Objective:**

The purpose of this study was to evaluate the safety and efficacy of mechanical TLE in female patients.

**Methods:**

A retrospective evaluation was performed on 3051 TLE patients (group 1: female; group 2: male) from a single tertiary referral center. All individuals received treatment using single sheath mechanical dilation and various venous approaches as required.

**Results:**

Our analysis included 3051 patients (group 1: 750; group 2: 2301), with a total of 5515 leads handled with removal. Female patients were younger, had a higher left ventricular ejection fraction, and lower prevalences of coronary artery disease and diabetes mellitus. Infection was more common in male patients, whereas lead malfunction or abandonment were more frequent in female patients. Radiologic success was lower in female patients (95.8% vs 97.5%; *P* = .003), but there was no significant difference in clinical success between groups (97.2% vs 97.5%; *P* = .872). However, major complications (1.33% vs 0.60%; *P* <.001) and procedural mortality (0.4% vs 0.1%; *P* <.001) were higher in females compared to male patients. After multivariate analysis, female sex emerged as the only predictor of major complications, including deaths (odds ratio 3.96; 95% confidence interval 1.39–11.24).

**Conclusion:**

TLE using unpowered simple mechanical sheaths in female patients is safe and effective, but is associated with lower radiologic success and higher complication rates and mortality than in males. This finding underscores the importance of recognizing sex differences in TLE outcomes.

## Graphical abstract

**Figure:**
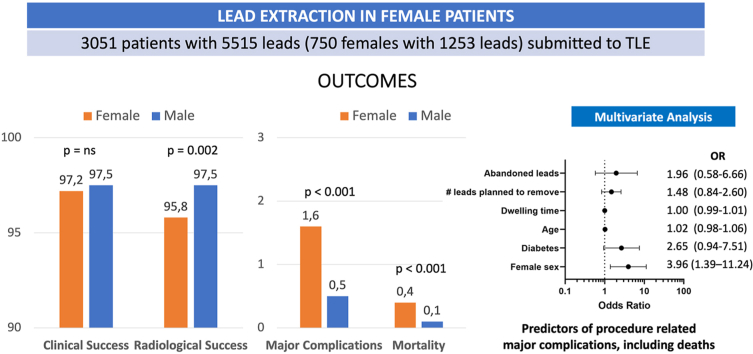

**Key Findings**

▪ Female patients demonstrated slightly lower rates of radiologic success with transvenous lead extraction (TLE) compared to males (95.8% vs 97.5%; *P* = .003).
▪ Despite the lower radiologic success, clinical success rates between female and male patients were not statistically different (97.2% vs 97.5%; *P* = .872).
▪ Female patients experienced higher rates of major complications (1.33% vs 0.60%; *P* <.001) and procedural mortality (0.4% vs 0.1%; *P* <.001).
▪ After multivariate analysis, female sex emerged as the only significant predictor of major complications, including death (odds ratio 3.96; 95% confidence 1.39–11.24).

## Introduction

Cardiac implantable electronic devices (CIEDs) play a crucial role in managing patients with various heart conditions, such as bradyarrhythmias, tachyarrhythmias, and heart failure. Over the past few decades, utilization of CIEDs has significantly increased, leading to a contextual increased need for transvenous lead extraction (TLE) procedures. TLE is essential for the management of patients with CIED-related complications, including infection, lead malfunction, and lead abandonment. Despite being a lifesaving procedure, TLE can be associated with potential risks and complications, which mandate a comprehensive understanding of the factors affecting its outcomes.

Sex differences have been reported to influence the outcomes of various cardiovascular procedures, including arrhythmia management and TLE.^1^, ^2^, ^3^, ^4^, ^5^, ^6^, ^7^, ^8^, ^9^, ^10^ It is pivotal to investigate whether sex differences also play a role in TLE outcomes, because this knowledge could help in optimizing TLE procedures for both male and female patients and could improve overall patient care in the management of CIED-related complications.

The present study aimed to evaluate the safety and efficacy of mechanical TLE in female patients. By comparing outcomes between female and male patients who underwent TLE at a single tertiary referral center, this study sought to provide insights into the potential sex differences in patient characteristics, risk factors, and complications related to TLE.

## Methods

We conducted a retrospective analysis of all consecutive patients who underwent TLE at our center from January 2009 to December 2022.

### Patients and procedure

All patients were enrolled at a tertiary referral center for TLE (Second Division of Cardiology, Pisa University Hospital, Pisa, Italy). Patients were divided into 2 groups according to sex: group 1 comprised female patients and group 2 comprised males. After obtaining informed consent, all patients were treated by an experienced operator in an electrophysiology laboratory, with an onsite surgical standby in most of the cases. Patients with an estimated high risk (ie, suspected perforation, high dwelling time of the leads, recalled leads) were treated by an electrophysiologist in the operating room, with the presence of surgical standby. No patient requiring TLE was excluded based on sex. Procedures were performed by a team of 4 experienced operators, and there was no difference in gender distribution among the patients treated by the different operators. The extraction technique has already been extensively described in the literature.^11^^,^^12^ The proximal end of the lead was clipped, and a standard stylet was inserted into the lead. Lead extraction was attempted through gentle manual traction, and, if unsuccessful, mechanical dilation was performed using a single sheath (ie, nontelescopic) technique. This typically involved inserting and moving multisized dilators (Cook Intravascular Inc, Leechburg, PA) through the venous entry site or alternatively through the right internal jugular or femoral vein, if required. In all cases, a temporary pacemaker and an arterial line for systemic pressure monitoring were placed. Procedural success, complications, indications, and lead characteristics were defined according to the 2018 EHRA Expert Consensus Statement on lead extraction.^13^ Radiologic success (for each lead) was considered when the lead was completely removed. Clinical success (considered for each patient) was defined as the complete removal of the leads or retention of a small portion of a lead that does not negatively impact the outcome goals of the procedure, with an absence of any permanently disabling complication or procedure-related death. A major complication was defined as any of the outcomes related to the procedure that is life-threatening or results in death (cardiac or noncardiac). A minor complication was defined as any undesired event related to the procedure that requires medical intervention or minor procedural intervention to remedy and does not persistently or significantly limit the patient's function, nor does it threaten life or cause death. The primary endpoint was the safety and efficacy of the procedure. Baseline clinical characteristics, procedural details, and survival data were obtained from the review of medical records, institutional databases, and phone interviews. The study was approved by the Institutional Review Board of the University Hospital of Pisa. The research reported in this article was conducted in strict accordance with ethical standards and guidelines to ensure the integrity, confidentiality, and protection of all participants involved. Specifically, this study adhered to the principles outlined in the Declaration of Helsinki for medical research involving human subjects.

### Statistical analysis

Data analysis was performed using SPSS (IBM SPSS Statistics for Windows, Version 25.0, IBM Corp, Armonk, NY). Continuous variables are reported as mean ± SD in the case of normal distribution, and median [interquartile range] in cases of non-Gaussian distribution. Categorical variables are expressed as percentage. Categorical variables were analyzed using the Pearson χ^2^ test. Continuous normal variables were compared using independent samples *T* test, and medians were compared using the Mann-Whitney *U* test. *P* <.05 was considered significant. Multivariate analysis was performed with a logistic regression model. *P* <.05 was considered significant.

## Results

### Patients

Overall, 3051 patients underwent TLE: 750 (24.6%) female (group 1) and 2301 (75.4%) male (group 2). Demographic characteristics are given in Table 1. Median age of the women was 67 years, and the median age of the men was slightly older at 70 years (*P* <.001). Notably, female patients had a lower prevalence of various comorbidities compared to their male counterparts. These included ischemic cardiomyopathy (12.9% vs 37.4%; *P* <.001); hypertension (47.3% vs 55.6%; *P* = .005); diabetes mellitus (16.7% vs 24.2%; *P* = .002); chronic heart failure (36.3% vs 52.2%; *P* <.001); chronic kidney disease (6% vs 16.7%; *P* <.001); and chronic obstructive pulmonary disease (7.6% vs 15.6%; *P* <.001). Finally, women had a lower number of implanted leads (2.14 ± 0.8 vs 2.32 ± 0.9; *P* = .002).

**Table 1:** Baseline patient characteristics

	Total (%)	Group 1 (female)	Group 2 (male)	*P* value
No. of patients (N)	3051	750	2301	
Age (y)	69 [59–76]	67 [51–76]	70 [60–76]	<.001
Age at first implant	62 [51–71]	60 [43.5–71]	63 [53–71]	<.001
Body mass index (kg/m^2^)	25.6 [23.5–28.0]	24.5 [21.7–27.3]	25.9 [24.1–28.3]	<.001
Body surface area (m^2^)	1.88 [1.76–2.01]	1.72 [1.61–1.81]	1.93 [1.82–2.05]	<.001
LVEF (%)	48 [35–58)	55 [45–60)	45 [33–55)	<.001
NYHA functional class III–IV	604 (19.8)	103 (13.8)	501 (21.8)	.001
Valvular implants	198 (6.5)	56 (7.5)	142 (6.2)	.489
Ischemic cardiomyopathy	958 (31.4)	97 (12.9)	861 (37.4)	<.001
Permanent atrial fibrillation	595 (19.5)	116 (15.5)	479 (20.8)	.023
Previous sternotomy	571 (18.7)	112 (14.9)	459 (19.9)	.030
Hypertension	1635 (53.6)	355 (47.3)	1280 (55.6)	.005
Diabetes mellitus	681 (22.3)	125 (16.7)	556 (24.2)	.002
Chronic heart failure	1474 (48.3)	272 (36.3)	1202 (52.2)	<.001
Chronic kidney disease	430 (14.1)	45 (6)	385 (16.7)	<.001
Chronic obstructive pulmonary disease	415 (13.6)	57 (7.6)	358 (15.6)	<.001
ICD	1141 (37.4)	202 (26.9)	939 (40.8)	<.001
CRT	1037 (34.0)	192 (25.6)	845 (36.7)	<.001
No. of leads per patient	2.24± 0.9	2.14 ± 0.8	2.32 ± 0.9	.002
Vegetations	485 (15.9)	92 (12.3)	393 (17.1)	.042
Large vegetations (>2 cm	107 (3.5)	11 (1.5)	96 (4.2)	.029
Anticoagulation	976 (32.0)	206 (27.5)	770 (33.5)	.055
Antiplatelet	978 (32.1)	151 (20.1)	827 (35.9)	<.001
Pacemaker-dependent	958 (31.4)	242 (32.3)	716 (31.1)	.737
One or more abandoned leads	629 (20.6)	118 (15.7)	511 (22.2)	.015

Values are given as median [interquartile range], n/N (%), or mean ± SD unless otherwise indicated.

CRT = cardiac resynchronization therapy; ICD = implantable cardioverter-defibrillator; LVEF = left ventricular ejection fraction; NYHA = New York Heart Association.

### Baseline lead characteristics

A total of 5515 leads were analyzed in this study: 1253 (22.7%) from female patients and 4262 (77.3%) from male patients. Leads characteristics are listed in Table 2. Average dwell time was longer for women (78.3 ± 74.3 months) than for men (72.7 ± 63.0 months) (*P* <.001). The distribution of leads varied between the 2 groups. Females had a lower proportion of implantable cardioverter-defibrillator (ICD) leads (16.5% vs 23.3%; *P* <.001), including both dual-coil (10.1% vs 15.9%; *P* <.001) and single-coil (6.4% vs 7.4%; *P* <.001) ICD leads. In contrast, the distribution of right ventricular pacing leads (40.8% in women and 33.5% in men; *P* = .800) and atrial leads (35.4% in women and 32.8% in men; *P* = .112) was relatively similar between the groups. However, coronary sinus leads were less common in women (7.3%) compared to men (10.4%) (*P* = .002).

**Table 2:** Baseline lead characteristics

	Total (%)	Group 1 (female)	Group 2 (male)	*P* value
No. of leads	5515	1253	4262	
Dwell time (mo) (mean ± SD)	73.9 ± 65.7	78.3 ± 74.3	72.7 ± 63.0	<.001
Atrial lead	1841 (33.4)	444 (35.4)	1397 (32.8)	.112
Right ventricular lead	1939 (35.1)	511 (40.8)	1428 (33.5)	.800
ICD lead	1200 (21.8)	206 (16.5)	994 (23.3)	<.001
Dual-coil ICD lead	802 (14.6)	126 (10.1)	676 (15.9)	<.001
Single-coil ICD lead	398 (7.2)	80 (6.4)	318 (7.4)	<.001
Coronary sinus lead	535 (9.7)	92 (7.3)	443 (10.4)	.002
Active fixation lead	1693 (30.7)	407 (32.5)	1286 (30.2)	.509

ICD = implantable cardioverter-defibrillator.

Indications for extraction varied between the 2 groups (Table 3). Infections were the primary reason for lead extraction, accounting for 69.9% of cases. Female patients had a lower proportion of infections (60.3%) than male patients (73.1%) (*P* <.001). Among the infections, systemic infections were more common in men (26.0%) than women (21.2%) (*P* = .009). Local infections also were more frequent in men (47.1%) compared to women (39.1%) (*P* <.001).

**Table 3:** Indication for the extraction

	Total (%)	Group 1 (female)	Group 2 (male)	*P* value
No. of patients (N)	3051	750	2301	
Infections	2133 (69.9)	452 (60.3)	1681 (73.1)	<.001
Systemic infections	756 (24.8)	159 (21.2)	597 (26.0)	.009
Local infectionsn/N (%)	1377 (45.1)	293 (39.1)	1084 (47.1)	<.001
No infection	918 (30.1)	298 (39.7)	620 (26.9)	<.001

Values are given as n/N (%) unless otherwise indicated.

### Procedures

**Figure 1:**
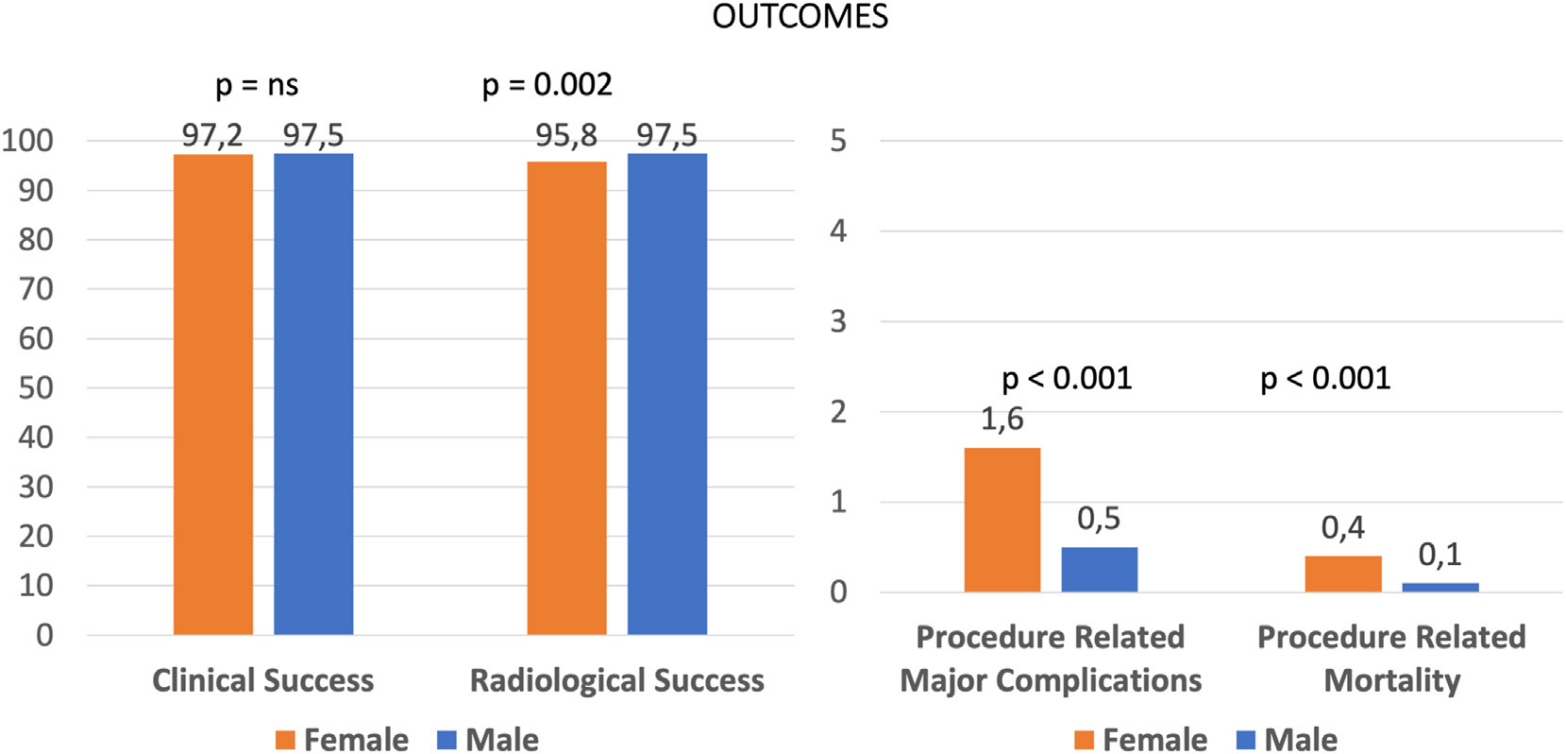
Radiologic success for leads, clinical success, procedure-related major complication rate, and mortality for patients according to different groups.

The overall success rate for complete lead extraction (radiologic success) was 97.2%, with a slightly lower rate for women (95.8%) compared to men (97.5%) (*P* = .002) (Figure 1 and Table 4). Manual traction proved to be more effective for female patients (18.7%) than for male patients (15.8%) (*P* = .003). The use of jugular or femoral approaches and the duration of fluoroscopy time showed no significant differences between groups (*P* = .966 and *P* = .425, respectively).

**Table 4:** Procedural results for leads

	Total (%)	Group 1 (female)	Group 2 (male)	*P* value
No. of leads (N)	5515	1253	4262	
Radiologic success (complete)	5358 (97.2)	1200 (95.8)	4158 (97.5)	.002
Manual traction effectiveness	908 (16.4)	234 (18.7)	674 (15.8)	.003
Jugular or femoral approach	572 (10.4)	130 (10.4)	442 (10.4)	.966
Fluoroscopy time (min))	15.0 [6.0–30.0]	14.0 [5.9–30.0]	15.0 [6.0–30.0]	.425

Values are given as n/N (%) or median [interquartile range] unless otherwise indicated.

There were no significant differences between females and males in terms of clinical success (97.2% vs 97.5%, respectively; *P* = .87) (Figure 1 and Table 5). However, the rates of major complications were more than 2× in female patients (1.33%) compared to male patients (0.6%) (*P* <.001), including a 4× higher rate of procedure-related deaths (0.4% vs 0.1%; *P* <.001). Minor complications also were more common in female patients (5.0%) than in male patients (3.4%) (*P* = .045). The overall rate of major and minor complications was higher in female patients (6.7%) compared to male patients (3.9%) (*P* = .019). In-hospital mortality before discharge was similar between the 2 groups (0.4% in both; *P* = .902).

**Table 5:** Procedural outcomes for patients

	Total (%)	Group 1 (female)	Group 2 (male)	*P* value
No. of patients (N)	3051	750	2301	
Clinical success	2972 (97.4)	729 (97.2)	2243 (97.5)	.872
Major complications	24 (0.8)	12 (1.6)	12 (0.5)	<.001
Death procedure-related (%)	6 (0.2)	3 (0.4)	3 (0.1)	<.001
Minor complications	117 (3.8)	38 (5.0)	79 (3.4)	.045
Major and minor complications	141 (4.6)	50 (6.7)	91 (3.9)	.019
In-hospital mortality before discharge	13 (0.4)	3 (0.4)	10 (0.4)	.902

Values are given as n/N (%) unless otherwise indicated.

### Predictors of adverse outcomes

**Figure 2:**
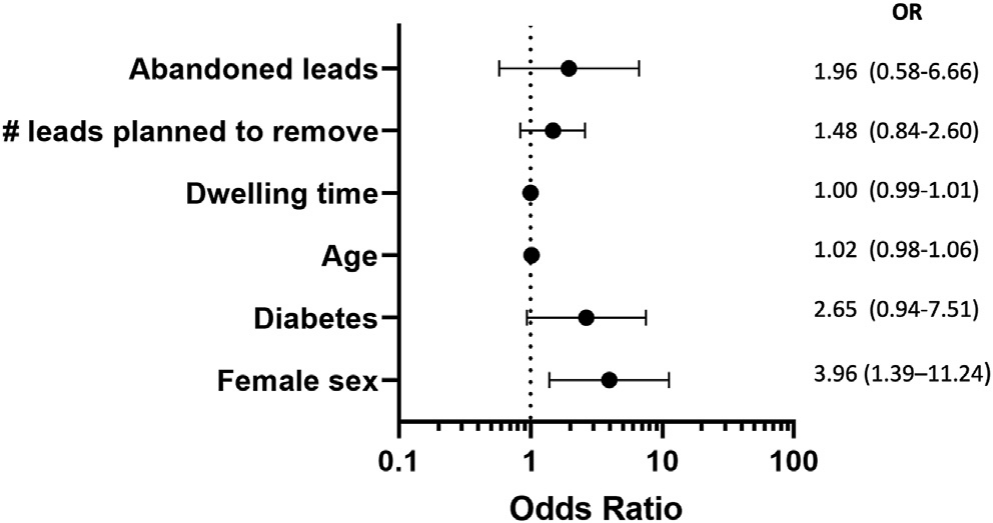
Predictors of procedure-related major complications, including deaths. OR = odds ratio.

Predictors of procedure-related major complications were checked, and significant risk factors are summarized in Table 6 and Figure 2. After multivariate analysis, female sex emerged as the only predictor of major complications, including deaths (odd ratio [OR] 3.96; 95% confidence interval [CI] 1.39–11.24).

**Table 6:** Univariate and multivariate logistic regression after stepwise algorithm selection on procedure-related major complications, including deaths

	Univariate analysis		Multivariate analysis	
	OR (95% CI)	*P* value	OR (95% CI)	*P* value
Patient age	1.04 (1.00–1.07)	.021	1.02 (0.98–1.06)	.330
Female sex	3.13 (1.40–7.00)	.006	3.96 (1.39–11.24)	.010
Body mass index	1.00 (0.97–1.04)	.775		
Creatinine	0.97 (0.69–1.38)	.822		
Hypertension	0.51 (0.18–1.48)	.199		
Diabetes	2.74 (1.01–7.41)	.056	2.65 (0.94–7.51)	.066
Ischemic heart disease	0.65 (0.18–2.38)	.501		
Previous cardiac surgery	0.28 (0.04–2.16)	.143		
LV ejection fraction (%)	1.02 (0.98–1.06)	.341		
Dwelling time	1.01 (1.00–1.01)	.001	1.00 (0.99–1.01)	.075
No. of cardiac leads	0.39 (0.85–1.75)	.821		
No. of leads planned for removal	1.69 (1.01–2.85)	.051	1.48 (0.84–2.60)	.179
Abandoned leads	3.13 (1.57–8.48)	.031	1.96 (0.58–6.66)	.282
Removal for infection	2.02 (0.69–5.94)	.167		
Venous occlusion	2.35 (0.52–10.65)	.263		
Active fixation lead	0.87 (0.32–2.37)	.790		
ICD lead	0.59 (0.23–1.52)	.263		
CRT device	0.49 (0.14–1.76)	.246		

CI = confidence interval; CRT = cardiac resynchronization therapy; ICD = implantable cardioverter-defibrillator; LV = left ventricle; OR = odds ratio.

## Discussion

This study explored differences between female and male patients in terms of baseline characteristics, lead types, and indications for extraction. Although the overall clinical success of TLE was similar between the 2 groups, female patients experienced a lower rate of radiologic success and a higher rate of complications. These findings suggest that sex-specific considerations should be taken into account when planning and performing TLE procedures to optimize patient outcomes.

Our analyses have indicated some compelling trends. For instance, we found that women in this study tended to be healthier with less coronary artery disease, improved ejection fraction, and less need for a defibrillator or biventricular ICD. Additionally, female patients were more likely to undergo lead extraction for abandoned and malfunctioning leads. It is plausible that fewer comorbidities, more abandoned leads coupled with longer dwell time, and smaller vascular diameter may have led to the development of more tenacious adhesions along the venous axis.

However, we also noted that the effectiveness of manual traction was slightly higher in women. This finding indicates that other factors, such as fewer ICD and more pacing leads, may have contributed to the outcome. In addition, the mean number of leads per patient was lower in females, which may have contributed to the higher success rate of manual traction. This suggests a complex interplay among patient characteristics, lead factors, and procedural outcomes that warrants further investigation.

The difference in radiologic success rates could be due to a variety of factors, including gender-specific differences in vascular diameter and wall thickness. A lower body surface area in women may explain the differences in vascular diameter and wall thickness. Furthermore, the significant difference in infection indication rates could also potentially explain the lower radiologic success rate in females. Noninfectious indications may have led the operator to terminate the procedure earlier, contributing to the lower radiologic success rate.

### Sex differences in patient characteristics and their implications

The observed sex differences in patient characteristics, such as age, ejection fraction, coronary artery disease, and diabetes mellitus prevalence, have potential implications for clinical practice. Because female patients undergoing TLE were mostly younger, they may present sex-specific differences in CIED indications and the overall prevalence of cardiovascular diseases.^2^ The higher ejection fraction in female patients is consistent with previous research demonstrating sex differences in cardiac function and arrhythmia presentation.^10^ These differences in cardiac function may influence the choice of device and lead selection. Indeed, the lower prevalence of coronary artery disease and diabetes mellitus in female patients may reflect well-established sex differences in cardiovascular disease risk factors and manifestations.^2^ Likewise, in our population, there were variations in lead types and implant characteristics between female and male patients; female patients were less likely to have ICD leads.^2^ Moreover, female patients had a lower percentage of coronary sinus leads. These differences may reflect disparities in arrhythmia presentation, underlying heart diseases, and management approaches.^10^

### Sex differences in indications for extraction and procedural data

In our population, infections were the primary indication for TLE in both female and male patients. However, the proportion of female patients with infections was significantly lower than that of male patients.^9^ Furthermore, female patients had a higher prevalence of noninfectious indications for TLE. These differences may affect the approach to TLE, including preprocedural planning, patient selection, and postprocedural care. Furthermore, understanding these differences may help identify high-risk patients, allowing for better risk stratification and more informed decision-making. The differences in indications for TLE between male and female patients may be related to underlying sex differences in CIED infection and lead malfunction rates. Linde et al^5^ noted that male patients had a higher risk of device-related infections, which could contribute to the higher infection rate observed in our analysis. Conversely, the higher rate of lead malfunction or abandonment in female patients could be attributed to sex-specific factors, such as unfavorable venous anatomy, which may predispose female patients to lead complications.^7^ Differences in procedural features between male and female patients, such as the number of leads per patient and dwell time, may be influenced by sex-specific factors, including patient age, comorbidities, and device indications. Understanding the factors contributing to these differences is crucial for optimizing patient outcomes and minimizing complications. For example, in cases in which the indication for lead extraction falls into class II and the leads are old with tenacious adhesions, it might be more appropriate in some instances to choose to abandon the lead *in situ* in female patients, thereby reducing potential procedural risks. Periprocedurally, knowing that female patients have a higher rate of complications could mean more intensive monitoring or follow-up for these patients.

### Sex differences in procedural outcomes

Our analysis revealed that female patients experienced lower complete procedural success rates and higher rates of major complications and procedural mortality compared to male patients. Radiologic success rates were slightly lower in female patients compared to male patients, consistent with previous studies.^4^^,^^7^ Although manual traction effectiveness was higher in female patients, a jugular or femoral approach showed no significant difference between the 2 groups. The lower rate of local and systemic infections in female patients may explain why, despite a lower radiologic success, the clinical success rates were similar between the 2 groups. However, female patients had higher incidences of major and minor complications.^3^^,^^8^^,^^14^ In a subanalysis of the ELECTRa (European Lead Extraction ConTRolled) study, cardiac avulsion or tear with tamponade was found to be more common in Riata lead extraction, particularly among female patients; with leads having a mean dwelling time >10 years; and when extracting multiple leads or requiring multiple sheaths.^3^^,^^8^^,^^14^ In our study, after multivariate analysis, female sex emerged as the only predictor of major complications (OR 3.96; 95% CI 1.39–11.24). In addition, female patients experienced a significantly higher rate of procedure-related deaths. These findings are consistent with previous studies, including those by Khalil et al,^9^ Jachec et al,^4^ and Bashir et al,^8^ which reported higher complication rates in female patients undergoing TLE. Potential explanations for these disparities include sex differences in venous anatomy, body habitus, and procedural technique.^4^ It is worth noting that an increased rate of major complications was witnessed in female patients even though our technique is known to allow for an overall generally safe approach. These findings emphasize the importance of recognizing female sex as a potential risk factor for complications and adopting tailored risk-mitigating strategies during TLE procedures.^1^^,^^6^ Given these findings, we suggest that guidelines and expert consensuses should weigh female sex as an important factor for procedural indication, especially for noninfective cases.

Bongiorni et al^3^ suggested that higher complication rates in female patients might be partly due to a lack of standardized protocols for managing sex-specific factors during TLE. The development of sex-specific guidelines and protocols may help reduce complications and improve outcomes in female patients. In addition, targeted training and education for clinicians regarding sex differences in TLE outcomes and their underlying mechanisms may contribute to better patient care.

Our study demonstrates notable differences in baseline characteristics, lead types, indications for extraction, and procedural outcomes between female and male patients undergoing TLE. Our findings, in the context of the literature, highlight the importance of acknowledging and addressing sex-specific differences in the management of cardiovascular diseases and TLE interventions. Furthermore, recognizing female sex as a potential risk factor for complications may help clinicians adopt tailored strategies to minimize adverse events and optimize patient outcomes during TLE procedures.^10^

### Study limitations and future directions

Our analysis has several limitations, including the retrospective nature of the study, the potential for selection bias, and the lack of long-term follow-up data. However, most of the studies published in the literature may have methodological limitations, such as small sample sizes or single-center designs, which could affect the generalizability of the results.

The proportion of infections among female patients was lower compared to male patients. This discrepancy might influence the final outcome because a more conservative approach typically is taken with noninfected catheters. Nonetheless, the observed increased complication rate among women raises the speculation that had there been more infectious indications among female patients, complication rates could have been even higher.

Future research should focus on prospective, multicenter studies with larger sample sizes to confirm and expand on the findings presented here. More effort is needed to elucidate the mechanisms underlying these differences and their potential impact on TLE outcomes. Long-term follow-up data would also provide valuable insights into the impact of sex differences on TLE outcomes over time. In addition, investigating the potential underlying biological and physiological mechanisms contributing to sex differences in TLE outcomes may improve patient care.

## Conclusion

TLE using unpowered simple mechanical sheaths in female patients is safe and effective, but is associated with lower radiologic success and higher complication rates and mortality than in male patients. This finding underscores the importance of recognizing sex differences in TLE outcomes.
